# Ammonia intercalated flower-like MoS_2_ nanosheet film as electrocatalyst for high efficient and stable hydrogen evolution

**DOI:** 10.1038/srep31092

**Published:** 2016-08-19

**Authors:** F. Z. Wang, M. J. Zheng, B. Zhang, C. Q. Zhu, Q. Li, L. Ma, W. Z. Shen

**Affiliations:** 1 Key Laboratory of Artificial Structure and Quantum Control, Ministry of Education, Department of Physics and Astronomy, Shanghai Jiao Tong University, Shanghai, 200240, PR China; 2 Collaborative Innovation Center of Advanced Microstructures, Nanjing University, Nanjing, 210093, PR China; 3 School of Chemistry and Chemical Technology, Shanghai Jiao Tong University, Shanghai, 200240, PR China

## Abstract

Ammonia intercalated flower-like MoS_2_ electrocatalyst film assembled by vertical orientated ultrathin nanosheet on graphite sheethas been successfully synthesized using one-step hydrothermal method. In this strategy, ammonia can effectively insert into the parallel plane of the MoS_2_ nanosheets, leading to the expansion of lattice and phase transfer from 2H to 1T, generating more active unsaturated sulfur atoms. The flower-like ammoniated MoS_2_ electrocatalysts with more active sites and large surface area exhibited excellent HER activity with a small Tafel slope and low onset overpotential, resulting a great enhancement in hydrogen evolution. The high efficient activity and recyclable utilization, as well as large-scale, indicate that it is a very promising electrocatalyst to replace Pt in industry application.

Nowadays, the environmental pollution caused by burning fossil fuels and the global energy crises have become more and more serious. As an ideal clean and sustainable fuel, hydrogen has been vigorously pursued as a promising candidate for replacing traditional petroleum fuels in the future^1^,^2^,^3^. And the electrocatalytic hydrogen evolution reaction (HER) is considered to be an important pathway for hydrogen production^4^,^5^. Noble metal catalysts, such as Pt, Ir and Au, are the most effective catalysts, but the cost and scarcity limit their wide application^6^,^7^. It remains a great challenge to develop non-noble metal HER catalysts exhibiting both high efficiency and stability.

Recently, transition metal phosphides^8^,^9^,^10^,^11^ and chalcogenides^12^,^13^,^14^,^15^ as the non-precious metal catalysts have shown a striking electrocatalytic performance for HER. Among these alternatives, two-dimensional (2D) layered material, molybdenum disulfide (MoS_2_) has received tremendous attention due to the earth-abundant composition, high activity and high chemical stability, leading to the development of various kinds of MoS_2_-based HER electrocatalysts^16^,^17^. During the past few years, both theoretical and experimental studies concluded that the HER activity arises from the sites located along the edges of the 2D MoS_2_ layer^18^,^19^,^20^,^21^. Hence, increasing the number of active sites is an efficient way to enhance the HER activity. Several efforts have been made to largely expose the edge sites for enhanced activity by reducing the dimension of MoS_2_ structures to the nanoscale or performing defect engineering^22^,^23^. Xie’s group has successful synthesized defect-rich MoS_2_ ultrathin nanosheets prepared by a facile hydrothermal method. With additional exposure of active edge sites for HER, the highly active HER catalysts showing an excellent activity with Tafel slopes of 50–55 mV/dec^24^,^25^. Cui’s group has grown vertically aligned MoS_2_ molecular layers on flat substrates. The vertically aligned MoS_2_ possess maximally exposed active edge sites. Through electrochemical intercalation of Li^+^ ion, the layer spacing, oxidation state, and the ratio of 2H semiconducting to 1T metallic phase can be continuously tuned resulting a dramatically improvement in HER activity^26^,^27^. In the 1T phase new active sites can be created on the basal planes due to crystal-strain. However, 1T-MoS_2_ was not stable and its synthetic process is relative complicated^28^,^29^,^30^.

Besides the aspect of active sites, the intrinsic resistance of catalysts is another crucial factor to affect the electrocatalytic activity because a high conductivity ensures a fast electron transport from conductive substrate to active site. Metal chalcogenide nanostructures fabricated on conductive substrates such as carbon nanotube^31^,^32^,^33^,^34^, carbon fiber^35^,^36^, F-doped tin oxide glass^37^ and Ti foils^38^ could decrease their resistances to some degree. Li’s grope has grown MoS_2_ nanocrystals on reduced graphene oxide^39^. With excellent electrical coupling to the underlying graphene network, the MoS_2_/RGO hybrid catalyst exhibited a relatively low onset potential and Tafel curve. However, the performance of HER electrocatalysts cannot be maximally enhanced through a single-objective optimization which is attributed to the contradictory relationship between active sites and conductivity. One step simultaneous structural and electronic modulations to increase both the active edge sites and the conductivity of MoS_2_ electrocatalysts still remain challenging.

In this paper, a flower-like ammoniated MoS_2_ ultrathin nanosheet array has been synthesized via a simple one-step hydrothermal method. Vertical orientated MoS_2_ nanosheet array can be grown uniformly on a graphite sheet. On one hand, ammonia in the reaction could effectively insert into the parallel plane of the MoS_2_ nanosheets, leading to the expansion of lattice and generation of 1T phase, generating more active unsaturated sulfur atoms in more disordered structure^24^. Furthermore, the dense nanosheet array structure increases electrodes surface area and be in favor of the escape of the hydrogen bubbles produced during the HER. On the other hand, conductivity through the parallel planes direction is much higher than the basal planes^22^. The vertical directly growth of MoS_2_ nanosheet arrays on graphite sheet can significantly decrease the resistance of the composite, inducing a high-quality, low-electrical-loss contact to the MoS_2_ electrocatalysts. These advantages make flower-like ammoniated MoS_2_ nanosheets array a highly competitive earth-abundant catalyst for HER.

## Results and Discussion

**Figure 1:**
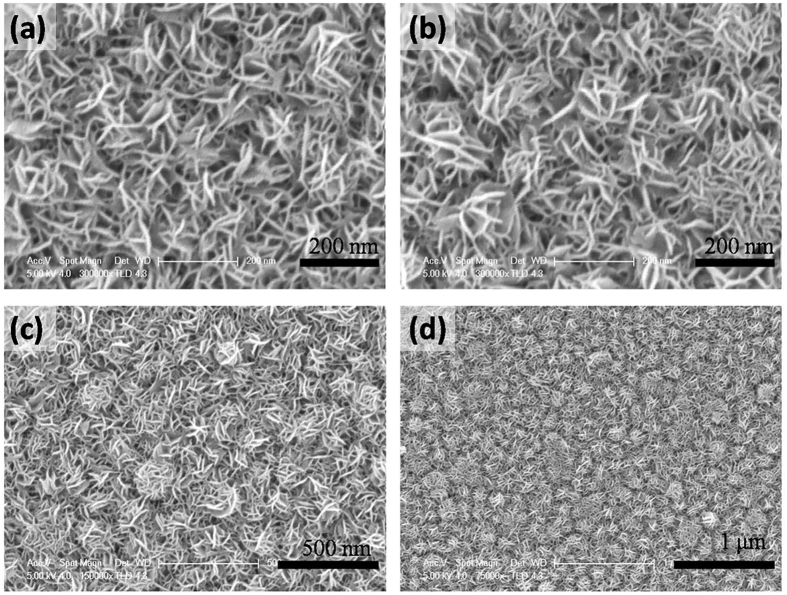
SEM micrographs with different magnifications of MoS_2_ flower-like nanosheet array structures.

**Figure 2:**
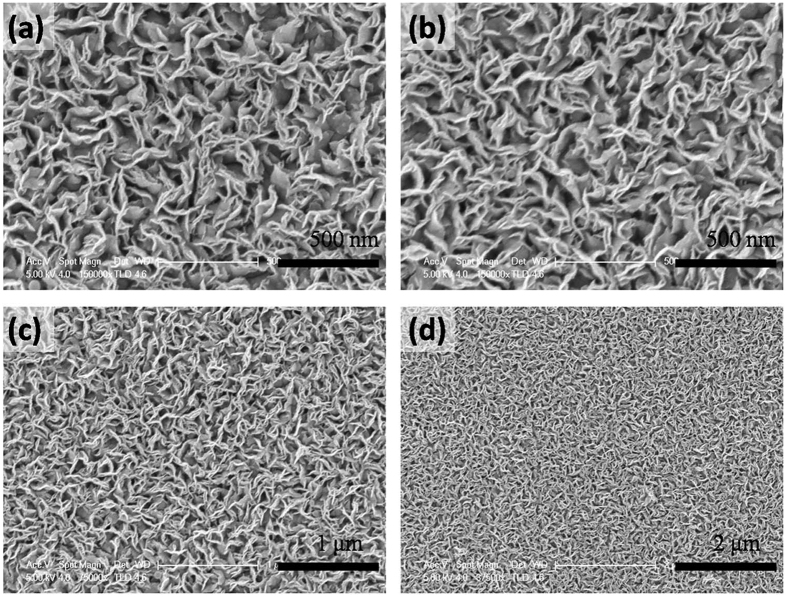
SEM micrographs with different magnifications showing uniform large size MoS_2_ nanosheet array structures.

**Figure 3:**
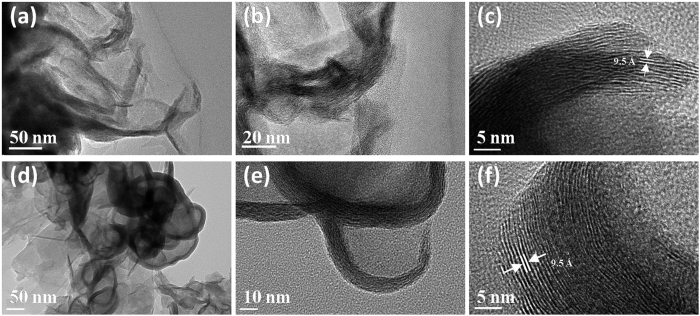
TEM micrographs of (**a**,**b**,**c**) FMNS and (**d,e,f**) MNS.

**Figure 4:**
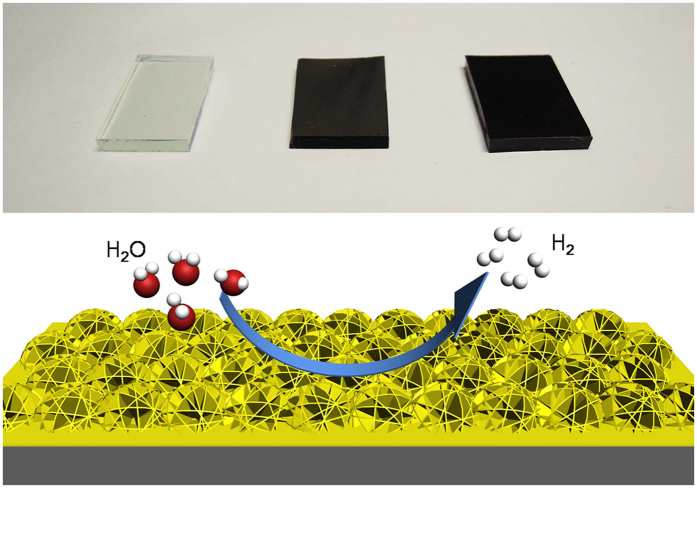
(**a**) Digital photos of a FTO glass substrate (left), a flower-like MoS_2_ nanosheet film on FTO (middle), and a large size MoS_2_ nanosheet film on FTO (right). Each substrate is about 1 × 2 cm^2^ in dimension. (**b**) Schematic representation of the flower-like structure in ammoniated MoS_2_ ultrathin nanosheets.

The ammoniated flower-like MoS_2_ nanosheet array is grown on the graphite sheet by a simple hydrothermal method. Generally, MoS_2_ obtained by a hydrothermal method tends to form sheet due to its intrinsic lamellar structures, and then these sheets aggregate together to form microspheres by van der Waals interaction and finally self-assembled into the three-dimensional nanostructures^40^,^41^,^42^. The scanning electron microscopy (SEM) images (Fig. 1) with different magnifications clearly shows that the well-defined flower-like morphology is preserved and the entire surface of the graphite sheet is uniformly covered by nanoflower like MoS_2_, where the diameter of the sphere is in the range of 200–300 nm. A high magnification view of MoS_2_ nanosheet decorated graphite reveals that the nanostructure consists of many ultrathin MoS_2_ nanosheet which grow vertically on the surface. Most of the nanosheets are in tight contact with each other, making the nanosheet film more stable, as illustrated in Fig. 1a,b. In order to further investigate the morphology effect on the HRE performance, a larger dimension MoS_2_ nanosheet array are also fabricated by a hydrothermal method in the ethanol-water mixture. The addition of ethanol can decrease the surface energy and allow their growth into large nanosheet products. A similar ordered nanosheet structure can be clearly observed in the low magnification SEM images (Fig. 2). The graphite sheet composite in the MoS_2_ nanosheet sample has a uniform diameter. The lateral length and the thickness in MNS are larger than FMNS. The microstructure of FMNS and MNS were further characterized by TEM. In good accordance with SEM results, the synthesized MoS_2_ electrodes show a few-layer nanosheet structure. The thickness of the nanosheets is about 10 nm. The distances of the (002) parallel lattice planes at the edge of MoS_2_ nanosheet in FMMS and MNS are about 9.5 Å, as shown in Fig. 3c,f. The large scale MoS_2_ nanosheet film grown on 1 × 2 cm^2^ FTO substrates are shown in Fig. 4.

**Figure 5:**
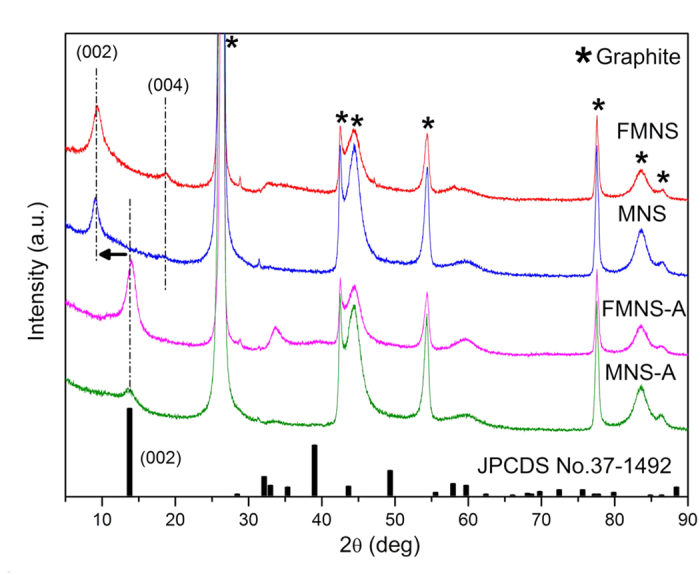
XRD patterns of the MoS_2_ nanosheet film samples.

The XRD patterns (Fig. 5) were performed on MoS_2_ catalyst samples to investigate the lamellar structure information. Besides the characteristic diffraction peaks assigned to (100), (102) and (110) of hexagonal MoS_2_ (JCPDS No. 37−1492), the new shifted peak (2θ = 9.3°) associated with the expanded (002) d spacing, and a second order diffraction peak (2θ = 18.6°) are observed in FMNS and MNS. Calculated by the Scherrer equation, the basal spacing increases by 3.4 Å which matches with the size of NH_3_/NH_4_^+^ ion whose diameter is ∼3.5 Å^43^,^44^. The interlayer space is consistent with the results from TEM images. When the samples were annealed at 500 °C for 2 h to remove the inserted ions, the XRD pattern is in agreement with the standard pristine MoS_2_. This reveals the phase transition to the 2H-MoS_2_, either on FMNS or MNS.

**Figure 6:**
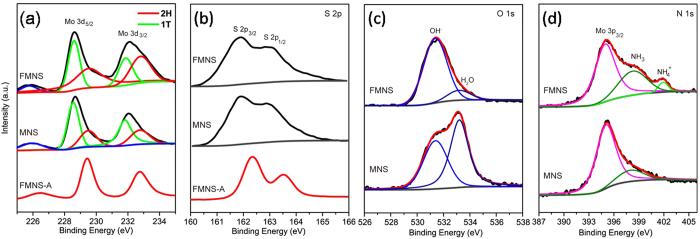
XPS spectra showing Mo 3d (**a**), S 2p (**b**), O 1 s (**c**) and N 1 s (**d**) peaks core level peak regions.

To characterize the chemical nature and bonding state of FMNS and MNS on GS surfaces, X-ray photoelectron spectroscopy (XPS) was employed. Figure 6 displays the detailed XPS scans for the Mo, S, O and N binding energies for the MoS_2_ catalysts. All of the spectra were calibrated by a carbon 1 s peak located at 284.50 eV. The 1T-MoS_2_ peaks were obtained after the deconvolution of the Mo 3d peaks in Fig. 6a. The Mo 3d5/2 and Mo 3d3/2 peaks shifted from ∼229.3 and ∼232.4 eV for the 2H-MoS_2_ of FMNS-A to ∼228.4 and∼231.5 eV for the 1T-MoS_2_, with a separation of binding energy at ∼0.9 eV^26^,^27^. The S 2p3/2 and 1/2 doublet peaks are also shown in Fig. 6b. In the high-resolution S 2p spectra, peaks at 162.3 and 163.5 eV correspond to the S^2−^ 2p3/2 and S^2−^ 2p1/2, and their separation energy is about 1.2 eV, which are typical characteristics of S^2−^ species^45^. Similarly downshift of bonding energies can be also observed in the S 2p peaks for FMNS and MNS samples. The shifts of Mo and S peaks indicate the metallic 1T phase in FMNS and MNS, consistent with previous reports^30^. The S 2p doublet peaks of the freshly prepared MoS_2_ exhibit broader peaks when compared with those of the annealed sample, indicating the existence of other binding signals, such as bridging S_2_^2−^ or apical S^2−^, which could result from the unsaturated S atoms and are known as active sites for the HER^46^,^47^,^48^. Thus better HER performance can be expected from the S-rich MoS_2_ sample. The O 1 s peak located at 531.4 and 533.2 eV are corresponding to the energy of oxygen in OH^−^ and H_2_O (Fig. 6c). Most importantly, the N1s spectrum of FMNS sample located at 398.3 and 402.0 eV demonstrates the existence of NH_4_^+^ and NH_3_ (Fig. 6d)^49^,^50^. However, different from FMNS, in MNS case, no NH_4_^+^ is observed, and the actual chemical composition should be (NH_3_)_x_MoS_2_. The enlargement of (002) planes can be ascribed to the ammonia intercalated into MoS_2_ during the synthesis process.

**Figure 7:**
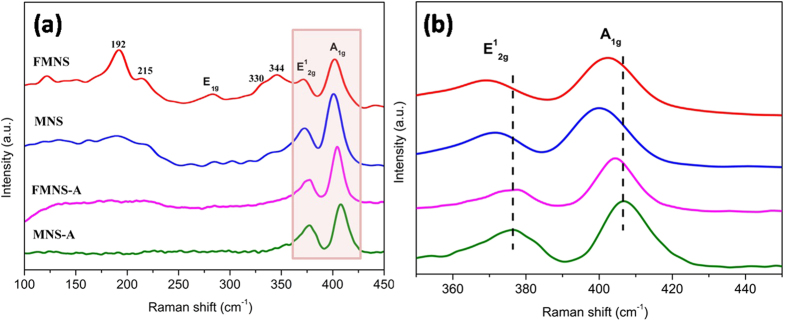
Raman spectra from FMNS-A, FMNS, MNS-A and MNS grown on graphite sheet.

Further insight into the nanostructure of FMNS and MNS are obtained by examination of Raman spectrum. As shown in Fig. 7a, the presence of 1T phase MoS_2_ of FMNS and MNS is confirmed by the Raman peaks emerging at 192, 215 and 340 cm^−1^ 26,27,28,30. The characteristic Raman shifts at about 378 and 403 cm^−1^ expected for the E^1^_2g_ and A_1g_ vibrational modes of hexagonal MoS_2_ are clearly observed in Fig. 7b. Meanwhile, the lower intensity of E^1^_2g_ peak compared with A_1g_ peak reveals the basal-edge-rich feature of the ultrathin MoS_2_ nanoplates. However, compared to the FMNS (403 cm^−1^), a red shift of A_1g_ (400 cm^−1^) is observed for the large size MoS_2_ nanosheet. When the layer number increases, the interlayer vander Waals force suppresses atom vibration, leading to higher force constants and blue shift of the A_1g_ mode. Therefore, the red shift of A_1g_ in MNS sample confirmed the lower stacking height of ultrathin MoS_2_ nanosheet. After the annealing process, the A_1g_ peaks in FMNS-A and MNS-A slightly shift to 404 cm^−1^, indicating an increase in stacking height^51^.

**Figure 8:**
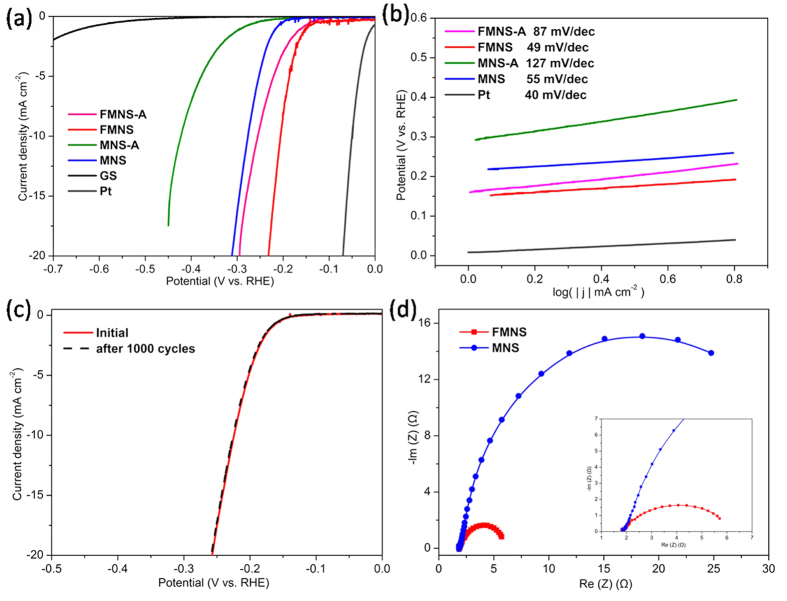
(**a**) Polarization curves of FMNS-A, FMNS, MNS-A, MNS and GS. The curves were recorded in N_2_-saturated 0.5 M H_2_SO_4_ solution at a scan rate of 0.5 mV/s. (**b**) Corresponding Tafel plots of FMNS-A, FMNS, MNS-A and MNS. (**c**) Polarization curves of the FMNS initially and after 1000 CV scanning between 0 and −0.3 V vs. RHE. (**d**) EIS spectra of FMNS and MNS with –0.2 V vs. RHE in N_2_-saturated 0.5 M H_2_SO_4_ electrolyte. Inset: corresponding EIS spectra at high frequency.

All of the above results clearly demonstrate that the vertically oriented and well interacting ammoniated MoS_2_ are established in a facile hydrothermal method, ultrathin MoS_2_ nanosheets *in situ* grown on the GS uniformly. Due to the higher surface area and electrical contact conductivity, the flower-like MoS_2_ may lead to a higher active electrocatalysis. To verify our hypothesis, the HER performance of the MoS_2_ nanosheet array samples were demonstrated without any binders in 0.5 M H_2_SO_4_ solution using a typical three-electrode setup, where the electrode was tested in a static state without rotation to mimic real industrial operation. Figure 8a shows the polarization curves with a low sweep rate of 0.5 mV/s. Compared to the GS, all the MoS_2_ nanosheet composites exhibit a relatively high HER activity. Among these samples, flower-like MoS_2_ nanosheet composites (FMNS and FMNS-A) exhibit an onset overpotential (*η*) of 120 mV, smaller than MNS and MNS-A (160 mV). The cathodic current rises rapidly at more negative potentials for all the samples. Notably, FMNS only requires an overpotential of 200 mV for driving a cathodic current density of 10 mA cm^−2^, smaller than those of FMNS-A (255 mV), MNS (276 mV), and MNS-A (422 mV). When the MoS_2_ nanosheet array was annealed in 500 °C, inducing a transition to a better crystalline structure, either on FMNS-A and MNS-A, the electrocatalytic performance dramatically decreased, as evidenced by their lower catalytic current densities.

The intrinsic HER kinetics of the above catalysts is shown by Tafel slope. The Tafel plots were fitted to the Tafel equation (η = a + blog|j|), where η is the over potential, a the Tafel constant, b the Tafel slope, and j the current density. As shown in Fig. 8b, the Tafel slope of flower-like MoS_2_ nanosheet (49 mV/dec) is the smallest among all the MoS_2_ nanosheet/GS composite. Thus, FMNS exhibits execellent HER activity. The apparent Tafel slope of the flower-like MoS_2_ nanosheet array is larger than that of the reported Pt based catalysts, but smaller than oxygen-incorporated MoS_2_ (55 mV/dec)^25^, defect-rich MoS_2_ (50 mV/dec)^24^ and exfoliated MoS_2_ (74 mV/dec)^52^. This significant improvement of the HER performance of the flower-like MoS_2_ sample is caused by the increased effective electrochemically active surface area enabled by the high density ultrathin nanosheet surface.

For the HER in acidic media, three principle steps for converting H^+^  to H_2_ have been suggested^39^,^53^,^54^.

Volmer discharge reaction:


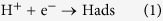


Heyrovsky desorption reaction:


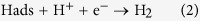


Tafel combination reaction:


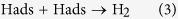


The Tafel slopes of about 120, 40, or 30 mV/dec will be achieved if the Volmer, Heyrovsky, or Tafel step is the rate-determining step, respectively. These values can be adopted as a guide in identifying the HER mechanisms. The slope falls within the range of 40–120 mV/dec, suggesting that the HER taking place on the GS surface would follow a Volmer-Heyrovsky mechanism, and that the rate of the discharge step would be consistent with that of the desorption step. To gain a better understanding of the interface reactions and electrode kinetics mechanism, electrochemical impedance spectroscopy (EIS) measurements were also performed. The Nyquist plots of the FMNS and MNS are given in Fig. 8d. The series resistances (Rs) observed for two samples (~1.7 Ω) comes from wiring and the electrolyte. The semicircular diameter in the EIS of the FMNS composites is much smaller than that of large size MoS_2_ nanosheet film, due to smaller charge transfer resistance (Rct). The higher Rct of the MNS can be attributed to the larger height of the MoS_2_ nanosheet arrays, long diffusion distance between the catalytic MoS_2_ edge sites and the substrate graphite, which will suppress the catalytic performance.

To rationalize this enhanced electrocatalytic performance, we measured the double layer capacitances (C_dl_) of these two electrodes. Cyclic voltammetry (CV) were performed at various scan rates in 0.1–0.2 V vs. RHE region (Fig. S1a,b), which could be mostly considered as the double-layer capacitive behavior. The double-layer capacitance is estimated by plotting the ∆*J* (*J*_a_-*J*_c_) at 0.15 V vs. RHE against the scan rate (Fig. S1c), which is expected to be linearly proportional to the effective surface area. The slope is twice C_dl_. The C_dl_ were calculated to be 41.4 mF for FMNS and 10.6 mF for MNS, respectively. The upper numbers of active sites of the FMNS and MNS samples were also estimated in N_2_-saturated 0.5 M H_2_SO_4_ solution through a simple cyclic voltammetry method (Fig. S2)^55^. The calculated numbers of the active sites of FMNS (18.3 × 10^−7^ mol cm^−2^) are higher than MNS (2.6 × 10^−7^ mol cm^−2^).

The outstanding HER electrocatalytic activity of the flower-like MoS_2_ nanosheet array can be attributed, on the one hand, to the morphology of the electrode that comprises high-density vertical orientated ultruthin MoS_2_ nanosheets, which maximizes the number of the exposed active sites and avoids the use of binders which would block active sites. On the other hand, the most unsaturated S atoms and the metallic 1T phase in freshly prepared FMNS contribute to the HER activity, given the fact that the catalytic activity of FMNS is higher than other samples.

To probe the stability of flower-like MoS_2_ nanosheet catalysts during HER process, a long-term cycling test was carried out. Figure 8c displays the polarization curves of ultrathin MoS_2_ nanoplate before and after 1000 cycles. After a long term-cycling, the catalyst shows similar polarization curve as before with negligible decay of current density, revealing the excellent stability of the nanosheet array film composites under HER conditions. Our flower-like nanosheets in tight contact with each other are robust enough to withstand a long-term cyclic voltammetry test. Small H_2_ bubbles generated on the rough surface can be timely released during the working process^38^, which in turn ensures a sufficient solid-liquid interface contact, resulting in a high electrocatalytic performance.

## Conclusion

In summary, flower-like ammoniated MoS_2_ nanosheet film grown on graphite sheet has been successfully developed via a simple hydrothermal method. The electrodes display excellent activity for hydrogen evolution with a small Tafel slope and more positive overpotential. The ammoniated dense flower-like vertical orientated ultrathin nanosheets not only increase the density of active sites but also could decrease the charge transfer resistance during HER process. The high active area and high electrical contact conductivity lead to better HER performance. Moreover, without any chemicals, the graphite sheet in the composite can be reused after polishing the electrode. Our results demonstrate that the flower-like MoS_2_ nanosheetcatalysts presented here are very promising for practical industry applications.

## Methods

### Synthesis of flower-like MoS_2_ nanosheet array

Flower-like MoS_2_ nanosheet array was directly grown on a graphite sheet (GS) by a hydrothermal method. 0.968 g sodiummolybdate (Na_2_MoO_4_ · 2H_2_O) and 0.7612 g thiourea (CH_4_N_2_S) were dissolved in 80 mL deionized water contained in a 100 mL Teflon-line stainless steel autoclave under vigorous stirring. Graphite sheet (1 × 1 cm^2^) was vertically immersed into the solution, and then the autoclave was sealed and maintained at 180 °C for 12 h. After cooling to room temperature, the flower-like MoS_2_ nanosheet array coated graphite was washed with deionized water and absolute ethanol, and dried in a vacuum oven at 60 °C for 12 h. The sample was denoted as FMNS.

For comparison, a large scale MoS_2_ nanosheet array was also synthesized. 0.06 g molybdenum oxide (MoO_3_), 0.07 g thioacetamide and 0.3 g urea were dispersed in 26 mL deionized water and 44 mL ethanol. Then, graphite sheet (1 × 1 cm^2^) was vertically immersed into the solution. The mixture was transferred to a 100 mL Teflon-line stainless steel autoclave and maintained at 180 °C for 12 h. After cooling to room temperature, the MoS_2_ nanosheet array coated graphite was washed with deionized water and absolute ethanol, and dried in a vacuum oven at 60 °C for 12 h. The sample was denoted as MNS.

The additional samples were annealed at 500 °C for 2 h in a high vacuum condition and denoted as FMNS-A and MNS-A.

### Materials characterizations

The morphology information was determined by a FEI Sirion 200 scanning electron microscope (SEM) and a JEOL 2100F transmission electron microscope (TEM). Samples were characterized by X-ray diffraction (XRD) by a Rigaku Ultima IV X-ray Diffractometer equipped with Cu Kα radiation. Surface composition of the sample was analyzed by X-ray photoelectron spectroscopy (XPS, AXIS ULTRA DLD, Kratos, Japan). Raman spectroscopy was recorded on Renishaw in Via-reflex system at room temperature. A laser wavelength of 532 nm was used as the excitation sources.

### Electrochemical Measurements

Photoelectrochemical measurements were performed using a PARSTAT 4000 workstation with a standard three-electrode system. Using the prepared samples as the working electrodes, a Pt gauze as the counter electrode, and Ag/AgCl as a reference electrode. Linear sweep voltammetry with scan rate of 0.5 mV/s was conducted in 0.5 M H_2_SO_4_. The electrochemical impedance spectroscopy (EIS) measurements for the FMNS and MNS were performed in N_2_-saturated 0.5 M H_2_SO_4_ solution with the frequencies range from 10 KHz to 0.1 Hz with an AC voltage of 5 mV. In all experiments, the electrolyte solutions were purged with N_2_ for 15 min prior to the experiments in order to remove oxygen. All the potentials reported in our manuscript were referenced to a reversible hydrogen electrode (RHE) by the Nernst equation *E*_RHE_ = *E*_Ag/AgCl_ + 0.059 pH + 0.197.

## Additional Information

**How to cite this article**: Wang, F. Z. *et al*. Ammonia intercalated flower-like MoS_2_ nanosheet film as electrocatalyst for high efficient and stable hydrogen evolution. *Sci. Rep.* **6**, 31092; doi: 10.1038/srep31092 (2016).

## Supplementary Material

Supplementary Information
