# Supplementary material for: Ammonia intercalated flower-like MoS2 nanosheet film as electrocatalyst for high efficient and stable hydrogen evolution
Source: Sci Rep. 2016 Aug 19;6:31092. doi: 10.1038/srep31092 (PMC4990931; doi:10.1038/srep31092)
Supplement: Supplementary Information [file srep31092-s1.doc]

**Ammonia intercalated flower-like MoS_2_ nanosheet film as electrocatalyst for high efficient and stable hydrogen evolution**

F. Z. Wang^1^, M. J. Zheng^1,2,*^, B. Zhang^1^, C. Q. Zhu^1^, Q. Li^1^, L. Ma^3^ & W. Z. Shen^1^

^1^Key Laboratory of Artificial Structure and Quantum Control, Ministry of Education, Department of Physics and Astronomy, Shanghai Jiao Tong University, Shanghai, 200240, PR China.

^2^Collaborative Innovation Center of Advanced Microstructures, Nanjing University, Nanjing, 210093, PR China.

^3^School of Chemistry and Chemical Technology, Shanghai Jiao Tong University, Shanghai, 200240, PR China.

^*^Corresponding author Fax: +86-021-54741040, E-mail: [mjzheng@sjtu.edu.cn](mailto:mjzheng@sjtu.edu.cn)


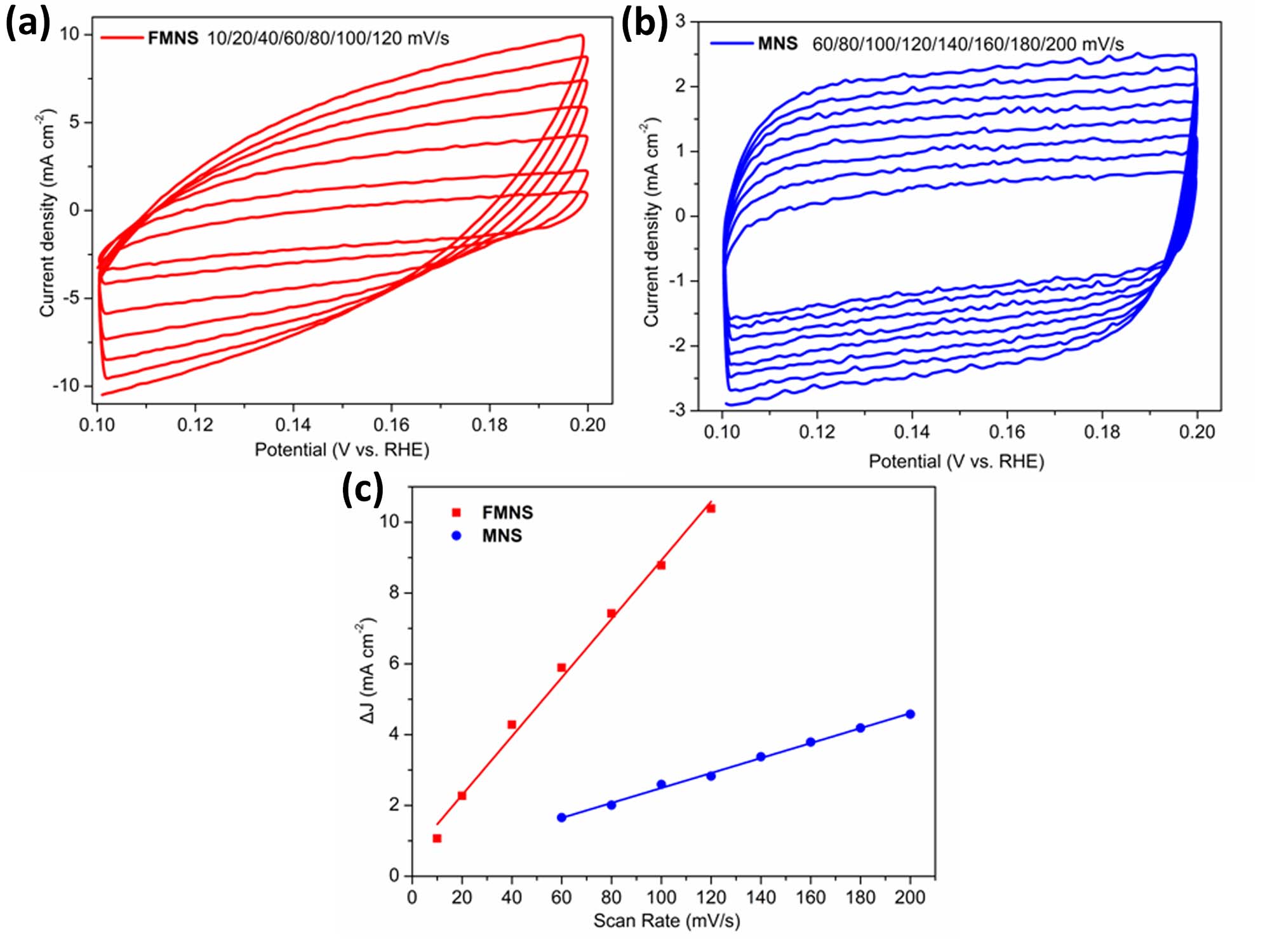


**Supplementary Figure 1**: Cyclic voltammograms in the region of 0.1–0.2 V vs. RHE for FMNS and MNS. The differences in current density (Δ*J*=*J*a-*J*c) at 0.15 V vs. RHE plotted against scan rate fitted to a linear regression allows for the estimation of C_dl_.


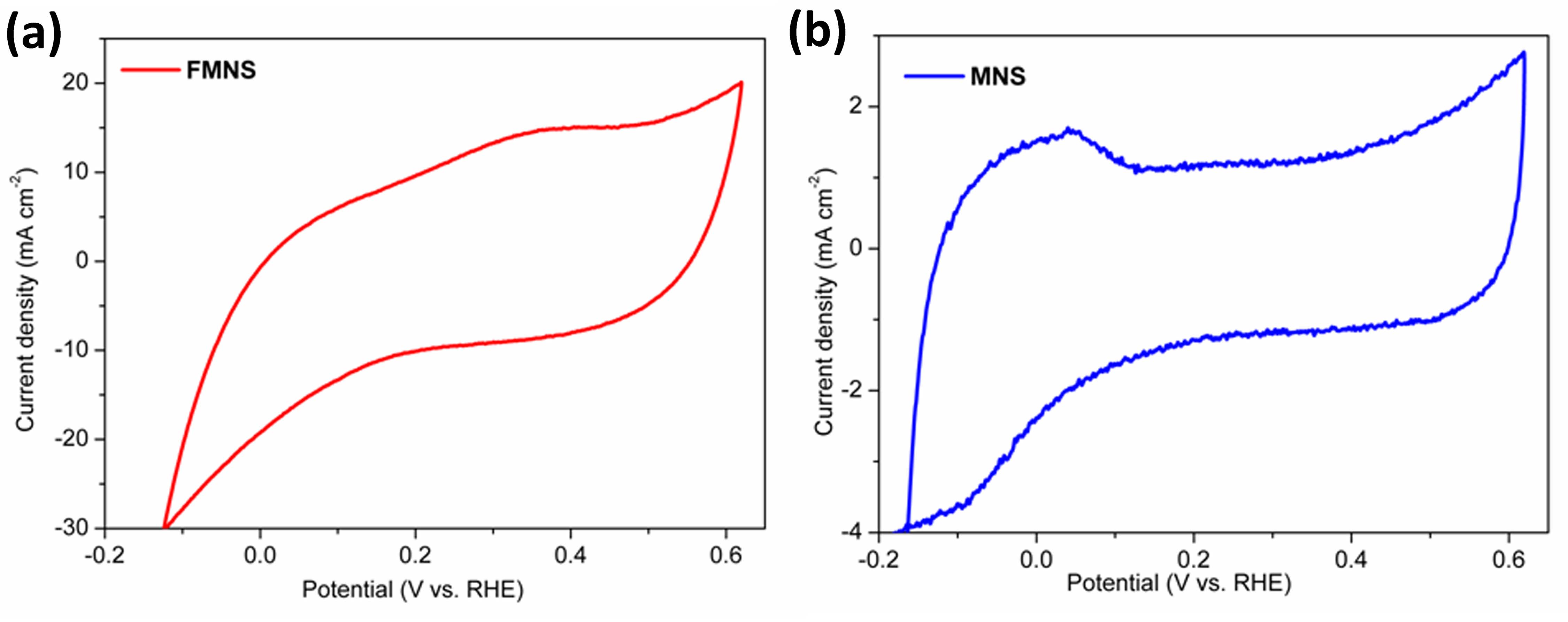


**Supplementary Figure 2**: Cyclic voltammograms (CV) in the region of –0.2 to 0.6 V vs. RHE for our FMNS and MNS in N_2_-saturated 0.5 M H_2_SO_4_ solution at a scan rate of 50 mV/s.
